# Complexities of cell-to-cell communication through membrane vesicles: implications for selective interaction of membrane vesicles with microbial cells

**DOI:** 10.3389/fmicb.2015.00633

**Published:** 2015-07-03

**Authors:** Yusuke Hasegawa, Hiroyuki Futamata, Yosuke Tashiro

**Affiliations:** Department of Applied Chemistry and Biochemical Engineering, Graduate School of Engineering, Shizuoka UniversityHamamatsu, Japan

**Keywords:** membrane vesicles, cell-to-cell communication, predation, horizontal gene transfer, quorum-sensing

## Introduction

Interaction between microbes in multicellular communities contributes to the development of complex microbial ecosystems. The secretion of various substances such as extracellular toxic compounds for combatting predation, extracellular DNA for horizontal gene transfer (HGT) and quorum-sensing (QS) signals for cell density-dependent cooperation, greatly influences microbial interactions (Hibbing et al., 2010; Tashiro et al., 2013). Study of the diffusion of substances secreted from donor cells to the extracellular environment and their uptake by recipient cells offers valuable insights for understanding microbial interactions.

**Figure 1:**
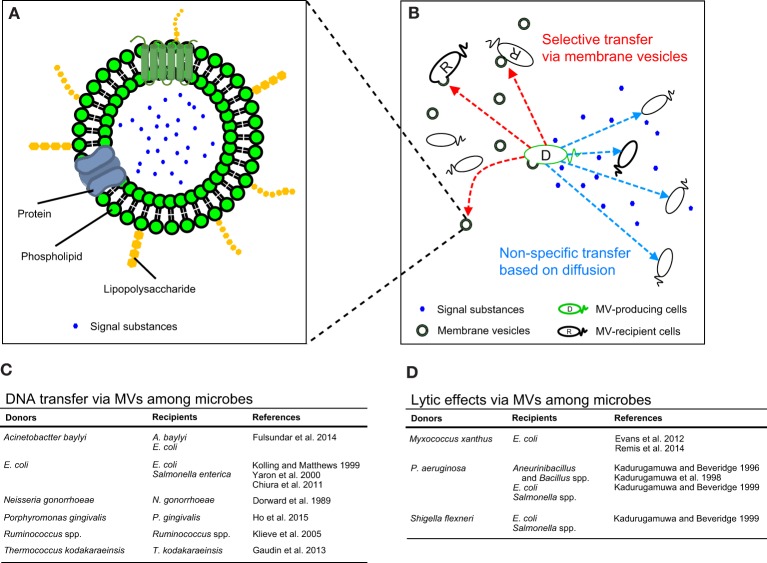
**(A)** The conceptual structure of membrane vesicles (MVs) derived from Gram-negative bacteria. MVs are composed of phospholipids, membrane proteins and lipopolysaccharides. In addition, various kinds of substances including DNA, exoproteins and quorum-sensing signals are contained in or are associated with MVs. **(B)** The pattern diagram of MV-mediated (red dashed arrows) and MV-independent (blue dashed arrows) cell-to-cell communications. While signals can be transmitted to bacterial cells non-specifically, MVs maintain the stability of MV-associated signals through protection from environmental stresses and may selectively deliver signals to target cells. **(C,D)** Representative interaction of MVs with microbial cells, including DNA transfer **(C)** and microbial lysis **(D)**.

The existence of membrane vesicles (MVs) increases the complexity involved in the diffusion of secreted substances during microbial interactions. MVs are extracellular particle-like liposome structures ranging from 20 to 200 nm in diameter (Figure 1A) and are pinched off from the external membrane of the microbe. The phenomenon of MV secretion has been observed in Gram-negative bacteria; however, recent studies have indicated that MVs are also produced by other prokaryotes including Gram-positive bacteria and archaea (Beveridge, 1999; Tashiro et al., 2010a, 2012; Haurat et al., 2015). MVs encapsulate membranal, periplasmic, and cytoplasmic components and play a role in the transfer of several compounds to organisms including both prokaryotic and eukaryotic cells. MVs contain proteins, DNA, RNA and in some cases, quorum sensing signals, and these substances are transferred to cells. Compared with freely-diffused chemical compounds secreted from bacteria, MVs have the following unique characteristics: (1) several chemical substances are highly concentrated in MVs, (2) interior substances in MVs are protected against environmental stresses, and (3) MVs play a role in effectively delivering these substances to cells. In this opinion article, we highlight the characteristics of MVs stated above and discuss the possibility of MV-mediated selective delivery to target cells (Figure 1B).

## Highly concentrated substances in MVs

The significant characteristic of MVs is their ability to encapsulate specific substances. Interior substances are maintained at high concentration and are protected from degradation by exterior stresses and enzymes. In the case of *Pseudomonas aeruginosa*, which is not only known as an opportunistic pathogen but also known to inhabit a variety of environments, a total of 68% of phospholipase C and 50% of alkaline phosphatase in the supernatant are localized in MVs along with the highly concentrated murein hydrolase (Kadurugamuwa and Beveridge, 1995, 1996). The encapsulation of toxic proteins provides an effective means for toxic transfer to not only eukaryotic cells but also other bacteria to counteract predation in the environment (Li et al., 1998; Evans et al., 2012). Furthermore, 86% of the *Pseudomonas* quinolone signal, which is one of the QS signals of *P. aeruginosa*, is localized in MVs, although smaller percentages (<1%) of other QS signals, including acyl-homoserine lactones, are localized in MVs (Mashburn and Whiteley, 2005). Such a highly concentrated QS signal is likely to effectively facilitate rapid alteration of gene expression in recipient cells. DNA is also highly concentrated in MVs, and MV-associated DNA is protected against extracellular DNase (Renelli et al., 2004). Several studies indicated that encapsulation of DNA contributes to HGT (Kolling and Matthews, 1999; Yaron et al., 2000; Fulsundar et al., 2014). Thus, the encapsulation of various signals in MVs plays an important role in microbial communications including QS and HGT, and increases effectiveness compared with diffusion-based interactions.

## Association of vesicles in eukaryotic cells

Whether MVs associate with a specific cellular surface remains unknown. In studies of eukaryotic intracellular vesicles, it has been shown that membrane-enveloped vesicles travel in between organelles in the cytoplasm, playing a role in transporting specific cargos to programmed locations via membrane fusion (Balch et al., 1984; Wilson et al., 1989; Sollner et al., 1993; Mcnew et al., 2000). In particular, a specific pairing between ligand and receptor allows vesicles to recognize the target compartment. In addition, the association between MVs and eukaryotic cells has been comprehensively studied in pathogenic bacteria, and MVs secreted from pathogens transfer virulent factors to cells (Parker et al., 2010; Chatterjee and Chaudhuri, 2011; Elmi et al., 2012; Rompikuntal et al., 2012; Bielaszewska et al., 2013). In particular, specific proteins localized on the surface of MVs increase the association with epithelial cells. For example, a heat-labile enterotoxin associated with MVs derived from enterotoxigenic *Escherichia coli* increases the association of MVs with cells (Kesty et al., 2004). The aminopeptidase of *P. aeruginosa* PaAP similarly increases the association of *P. aeruginosa* MVs with lung epithelial cells (Bauman and Kuehn, 2009). In addition, bacterial cytotoxin VacA increases the adhesion of MVs with cells, most likely by increasing the association of MV lipopolysaccharide with cells (Parker et al., 2010). Thus, MVs can contribute to the virulence of eukaryotic cells, but little is understood regarding whether such associations are promoted by specific interactions between a ligand and a receptor. Understanding the specific association of MVs with cells would enable the development of applications involving MVs as vehicles for cell-specific drug delivery (Gujrati et al., 2014).

## Selective association between MVs and microbial cells

The association of MVs and microbial cells has been a subject of several studies on electric charge and hydrophobicity of cellular surfaces. The surfaces of bacterial cells are usually negatively charged and rich in divalent cations that stabilize surface charges such as Mg^2+^ or Ca^2+^. When freely floating MVs encounter bacterial cells, these divalent cations act as a bridge between the negative charged surfaces, and the adhesion of negatively charged MVs on a bacterial cellular surface is stabilized (Kadurugamuwa and Beveridge, 1996; Tashiro et al., 2010b).

Hydrophobicity of a cellular surface is also an important factor for adhesion of MVs on bacterial cells. It has been shown that *P. aeruginosa* MVs can more easily attach to the hydrophilic surface of *Bacillus subtilis* than to other Gram-positive bacteria (Macdonald and Beveridge, 2002), indicating that liberated MVs from bacterial cells can selectively interact with bacterial cells. Nakao et al. developed a novel method to purify MVs derived from *Porphyromonas gingivalis* using epoxy-coated magnetic beads (Nakao et al., 2014) and the authors suggested that binding between MVs and epoxy beads is facilitated via a hydrophobic interaction.

In another report, MVs derived from *Myxococcus xanthus* adhered not only to the cells but also to MVs to form vesicle chains between cells (Remis et al., 2014). The detailed mechanism of the connection has not yet been elucidated, but it has been suggested that lipopolysaccharides (LPS) plays a role in MV-cell recognition because the distance between MVs and cellular surface was 5–10 nm, corresponding to the LPS.

Thus, MVs secreted from bacteria have characteristic surfaces and possess variable potential to attach to certain surfaces. Because specific proteins are selectively assimilated into MVs from bacterial cells, it is possible that the association of MVs with bacterial cells is a highly specific process based on a specific ligand-receptor interaction.

## Transfer of MV interior substances to microbial cells

The importance of the contents of MVs has been exemplified by the critical role the transported material plays in the transfer of DNA (Figure 1C) (Dorward et al., 1989; Kolling and Matthews, 1999; Yaron et al., 2000; Klieve et al., 2005; Chiura et al., 2011; Gaudin et al., 2013; Fulsundar et al., 2014; Ho et al., 2015), bacterial lysis (Figure 1D) (Kadurugamuwa and Beveridge, 1996, 1999; Kadurugamuwa et al., 1998; Li et al., 1998; Evans et al., 2012; Remis et al., 2014) and QS-regulated gene expression (Tashiro et al., 2010b). It should be noted that membrane fusion does not always occur when interior substances in MVs are transferred to bacterial cells.

With regard to DNA transfer via MVs, the adhesion of MVs with bacterial cellular surface but not membrane fusion has been confirmed by transmission electron microscope observation in *E. coli* O157:H7 (Kolling and Matthews, 1999). Recently, however, it has been shown that MVs are integrated to recipient cells and DNA is transferred from *Acinetobacter baylyi* to *E. coli* and *A. baylyi* (Fulsundar et al., 2014).

Microbial predation using MVs occurs when virulent factors or peptidoglycan hydrolytic enzymes contained in MVs are transferred to other bacterial cells. It has been suggested that the mechanism of bacterial lysis via MVs secreted from Gram-negative bacteria differs in whether recipient cells are Gram-negative or positive. MVs derived from *P. aeruginosa* can attach to the surface of *E. coli* and *Staphylococcus aureus*, while they are able to fuse with *E. coli* but not *S. aureus* (Kadurugamuwa and Beveridge, 1996). The authors have suggested a possible mechanism described below. Because MVs have high curvature, negatively charged O-side chains of LPS are loosely packaged and they could form salt-bridging by cations such as Ca^2+^ and Mg^2+^, with bacterial surfaces on which such cations are rich. For the association of MVs with a Gram-positive bacterial surface, this event would break apart the high curvature of MVs, and thereby open MVs, resulting in the liberation of interior lytic enzymes and the digestion of the cell wall. This event would enable the transition of the content from MVs to cells, possibly through permeation of the cellular membrane without fusion. On the other hand, MVs fuse into outer membrane of Gram-negative bacteria because they possess a compatible bilayer surface. Thus, it is considered that selective MV integration with recipient cells occurs, but interior substances in MVs can be transferred to microbial cells even when MVs just adhere on the cellular surfaces.

## Concluding remarks and perspectives

Therefore, MVs encapsulate signaling substances secreted from cells, protect these substances from environmental stresses and maintain these substances at high concentrations. In addition, signals associated with MVs are likely transferred to specific bacterial cells. Such signal transfer via MVs is considerably different from diffusion-based cell-to-cell communication (Figure 1B). The transfer of substances via MVs increases accuracy, swiftness and effectiveness of responses in combating predation, HGT and QS. Thus, cell-to-cell communications are comprised of not only a simple method based on diffusive substances but also very complicated aspects. Microbes intricately communicate through as-yet-unknown methods using MVs, thereby influencing interspecies networks, microbial community organization and ecosystem dynamics.

### Conflict of interest statement

The authors declare that the research was conducted in the absence of any commercial or financial relationships that could be construed as a potential conflict of interest.

## References

[B1] Balch W. E. Glick B. S. Rothman J. E. (1984). Sequential intermediates in the pathway of intercompartmental transport in a cell-free system. Cell 39, 525–536. 10.1016/0092-8674(84)90459-8 6096009

[B2] Bauman S. J. Kuehn M. J. (2009). *Pseudomonas aeruginosa* vesicles associate with and are internalized by human lung epithelial cells. BMC Microbiol 9:26. 10.1186/1471-2180-9-26 19192306 PMC2653510

[B3] Beveridge T. (1999). Structures of gram-negative cell walls and their derived membrane vesicles. J. Bacteriol. 181, 4725–4733. 10438737 10.1128/jb.181.16.4725-4733.1999 PMC93954

[B4] Bielaszewska M. Rüter C. Kunsmann L. Greune L. Bauwens A. Zhang W. (2013). Enterohemorrhagic *Escherichia coli* hemolysin employs outer membrane vesicles to target mitochondria and cause endothelial and epithelial apoptosis. PLoS Pathog. 9:e1003797 10.1371/journal.ppat.1003797 24348251 PMC3861543

[B5] Chatterjee D. Chaudhuri K. (2011). Association of cholera toxin with *Vibrio cholerae* outer membrane vesicles which are internalized by human intestinal epithelial cells. FEBS Lett. 585, 1357–1362. 10.1016/j.febslet.2011.04.017 21510946

[B6] Chiura H. X. Kogure K. Hagemann S. Ellinger A. Velimirov B. (2011). Evidence for particle-induced horizontal gene transfer and serial transduction between bacteria. FEMS Microbial. Ecol. 76, 576–591. 10.1111/j.1574-6941.2011.01077.x 21361998

[B7] Dorward D. W. Garon C. F. Judd R. C. (1989). Export and intercellular transfer of DNA via membrane blebs of *Neisseria gonorrhoeae*. J. Bacteriol. 171, 2499–2505. 2496108 10.1128/jb.171.5.2499-2505.1989 PMC209926

[B8] Elmi A. Watson E. Sandu P. Gundogdu O. Mills D. C. Inglis N. F.. (2012). Campylobacter jejuni outer membrane vesicles play an important role in bacterial interactions with human intestinal epithelial cells. Infect. Immun. 80, 4089–4098. 10.1128/IAI.00161-12 22966047 PMC3497446

[B9] Evans A. G. L. Davey H. M. Cookson A. Currinn H. Cooke-Fox G. Stanczyk P. J.. (2012). Predatory activity of *Myxococcus xanthus* outer-membrane vesicles and properties of their hydrolase cargo. Microbiology 158, 2742–2752. 10.1099/mic.0.060343-0 22977088

[B10] Fulsundar S. Harms K. Flaten G. E. Johnsen P. J. Chopade B. A. Nielsen K. M. (2014). Gene transfer potential of outer membrane vesicles of *Acinetobacter baylyi* and effects of stress on vesiculation. Appl. Environ. Microbiol. 80, 3469–3483. 10.1128/AEM.04248-13 24657872 PMC4018862

[B11] Gaudin M. Gauliard E. Schouten S. Houel-Renault L. Lenormand P. Marguet E.. (2013). Hyperthermophilic archaea produce membrane vesicles that can transfer DNA. Environ. Microbiol. Rep. 5, 109–116. 10.1111/j.1758-2229.2012.00348.x 23757139

[B12] Gujrati V. Kim S. Kim S.-H. Min J. J. Choy H. E. Kim S. C.. (2014). Bioengineered bacterial outer membrane vesicles as cell-specific drug-delivery vehicles for cancer therapy. ACS Nano 8, 1525–1537. 10.1021/nn405724x 24410085

[B13] Haurat M. F. Elhenawy W. Feldman Mario F. (2015). Prokaryotic membrane vesicles: new insights on biogenesis and biological roles. Biol. Chem. 396, 95. 10.1515/hsz-2014-0183 25178905

[B14] Hibbing M. E. Fuqua C. Parsek M. R. Peterson S. B. (2010). Bacterial competition: surviving and thriving in the microbial jungle. Nat. Rev. Microbiol. 8, 15–25. 10.1038/nrmicro2259 19946288 PMC2879262

[B15] Ho M.-H. Chen C.-H. Goodwin J. S. Wang B.-Y. Xie H. (2015). Functional advantages of *Porphyromonas gingivalis* vesicles. PLoS ONE 10:e0123448. 10.1371/journal.pone.0123448 25897780 PMC4405273

[B16] Kadurugamuwa J. Mayer A. Messner P. Sára M. Sleytr U. Beveridge T. (1998). S-layered Aneurinibacillus and Bacillus spp. are susceptible to the lytic action of *Pseudomonas aeruginosa* membrane vesicles. J. Bacteriol. 180, 2306–2311. 9573179 10.1128/jb.180.9.2306-2311.1998 PMC107169

[B17] Kadurugamuwa J. L. Beveridge T. J. (1995). Virulence factors are released from *Pseudomonas aeruginosa* in association with membrane vesicles during normal growth and exposure to gentamicin: a novel mechanism of enzyme secretion. J. Bacteriol. 177, 3998–4008. 7608073 10.1128/jb.177.14.3998-4008.1995 PMC177130

[B18] Kadurugamuwa J. L. Beveridge T. J. (1996). Bacteriolytic effect of membrane vesicles from *Pseudomonas aeruginosa* on other bacteria including pathogens: conceptually new antibiotics. J. Bacteriol. 178, 2767–2774. 8631663 10.1128/jb.178.10.2767-2774.1996 PMC178010

[B19] Kadurugamuwa J. L. Beveridge T. J. (1999). Membrane vesicles derived from *Pseudomonas aeruginosa* and *Shigella flexneri* can be integrated into the surfaces of other gram-negative bacteria. Microbiology 145, 2051–2060. 10.1099/13500872-145-8-2051 10463171

[B20] Kesty N. Mason K. Reedy M. Miller S. Kuehn M. (2004). Enterotoxigenic *Escherichia coli* vesicles target toxin delivery into mammalian cells. EMBO J. 23, 4538–4549. 10.1038/sj.emboj.7600471 15549136 PMC533055

[B21] Klieve A. V. Yokoyama M. T. Forster R. J. Ouwerkerk D. Bain P. A. Mawhinney E. L. (2005). Naturally occurring DNA transfer system associated with membrane vesicles in cellulolytic *Ruminococcus* spp. of ruminal origin. Appl. Environ. Microbiol. 71, 4248–4253. 10.1128/AEM.71.8.4248-4253.2005 16085810 PMC1183309

[B22] Kolling G. L. Matthews K. R. (1999). Export of virulence genes and Shiga toxin by membrane vesicles of *Escherichia coli* O157:H7. Appl. Environ. Microbiol. 65, 1843–1848. 10223967 10.1128/aem.65.5.1843-1848.1999 PMC91264

[B23] Li Z. Clarke A. J. Beveridge T. J. (1998). Gram-negative bacteria produce membrane vesicles which are capable of killing other bacteria. J. Bacteriol. 180, 5478–5483. 9765585 10.1128/jb.180.20.5478-5483.1998 PMC107602

[B24] Macdonald K. L. Beveridge T. J. (2002). Bactericidal effect of gentamicin-induced membrane vesicles derived from *Pseudomonas aeruginosa* PAO1 on gram-positive bacteria. Can. J. Microbiol. 48, 810–820. 10.1139/w02-077 12455613

[B25] Mashburn L. M. Whiteley M. (2005). Membrane vesicles traffic signals and facilitate group activities in a prokaryote. Nature 437, 422–425. 10.1038/nature03925 16163359

[B26] Mcnew J. A. Parlati F. Fukuda R. Johnston R. J. Paz K. Paumet F.. (2000). Compartmental specificity of cellular membrane fusion encoded in SNARE proteins. Nature 407, 153–159. 10.1038/35025000 11001046

[B27] Nakao R. Kikushima K. Higuchi H. Obana N. Nomura N. Bai D. (2014). A novel approach for purification and selective capture of membrane vesicles of the periodontopathic bacterium, *Porphyromonas gingivalis*: membrane vesicles bind to magnetic beads coated with epoxy groups in a noncovalent, species-specific manner. PLoS ONE 9:e95137 10.1371/journal.pone.0095137 24830438 PMC4022494

[B28] Parker H. Chitcholtan K. Hampton M. B. Keenan J. I. (2010). Uptake of *Helicobacter pylori* outer membrane vesicles by gastric epithelial cells. Infect. Immun. 78, 5054–5061. 10.1128/IAI.00299-10 20876296 PMC2981328

[B29] Remis J. P. Wei D. Gorur A. Zemla M. Haraga J. Allen S.. (2014). Bacterial social networks: structure and composition of *Myxococcus xanthus* outer membrane vesicle chains. Environ. Microbiol. 16, 598–610. 10.1111/1462-2920.12187 23848955 PMC4234120

[B30] Renelli M. Matias V. Lo R. Y. Beveridge T. J. (2004). DNA-containing membrane vesicles of *Pseudomonas aeruginosa* PAO1 and their genetic transformation potential. Microbiology 150, 2161–2169. 10.1099/mic.0.26841-0 15256559

[B31] Rompikuntal P. K. Thay B. Khan M. K. Alanko J. Penttinen A.-M. Asikainen S.. (2012). Perinuclear localization of internalized outer membrane vesicles carrying active cytolethal distending toxin from *Aggregatibacter actinomycetemcomitans*. Infect. Immun. 80, 31–42. 10.1128/IAI.06069-11 22025516 PMC3255663

[B32] Sollner T. Whiteheart S. W. Brunner M. Erdjument-Bromage H. Geromanos S. Tempst P.. (1993). SNAP receptors implicated in vesicle targeting and fusion. Nature 362, 318–324. 10.1038/362318a0 8455717

[B33] Tashiro Y. Ichikawa S. Nakajima-Kambe T. Uchiyama H. Nomura N. (2010a). *Pseudomonas* quinolone signal affects membrane vesicle production in not only Gram-negative but also Gram-positive bacteria. Microb. Environ. 25, 120–125. 10.1264/jsme2.ME09182 21576862

[B34] Tashiro Y. Ichikawa S. Shimizu M. Toyofuku M. Takaya N. Nakajima-Kambe T.. (2010b). Variation of physiochemical properties and cell association activity of membrane vesicles with growth phase in *Pseudomonas aeruginosa*. Appl. Environ. Microbiol. 76, 3732–3739. 10.1128/AEM.02794-09 20382806 PMC2876431

[B35] Tashiro Y. Uchiyama H. Nomura N. (2012). Multifunctional membrane vesicles in *Pseudomonas aeruginosa*. Environ. Microbiol. 14, 1349–1362. 10.1111/j.1462-2920.2011.02632.x 22103313

[B36] Tashiro Y. Yawata Y. Toyofuku M. Uchiyama H. Nomura N. (2013). Interspecies interaction between *Pseudomonas aeruginosa* and other microorganisms. Microb. Environ. 28, 13–24. 10.1264/jsme2.ME12167 23363620 PMC4070684

[B37] Wilson D. W. Wilcox C. A. Flynn G. C. Chen E. Kuang W.-J. Henzel W. J.. (1989). A fusion protein required for vesicle-mediated transport in both mammalian cells and yeast. Nature 339, 355–359. 10.1038/339355a0 2657434

[B38] Yaron S. Kolling G. Simon L. Matthews K. (2000). Vesicle-mediated transfer of virulence genes from *Escherichia coli* O157:H7 to other enteric bacteria. Appl. Environ. Microbiol. 66, 4414–4420. 10.1128/AEM.66.10.4414-4420.2000 11010892 PMC92318

